# The migration of placenta even after 30 gestational weeks is a risk factor for postpartum hemorrhage

**DOI:** 10.1016/j.xagr.2025.100593

**Published:** 2025-11-30

**Authors:** Gaku Yamamoto, Kosuke Hiramatsu, Yoko Kawanishi, Mamoru Kakuda, Koji Nakamura, Tatsuya Miyake, Kazuya Mimura, Toshihiro Kimura, Masayuki Endo, Tadashi Kimura, Michiko Kodama

**Affiliations:** 1 Department of Obstetrics and Gynecology, Osaka University Hospital, Suita, Osaka, Japan (Yamamoto, Hiramatsu, Kawanishi, Kakuda, Nakamura, Miyake, Mimura, Toshihiro Kimura, Endo, Tadashi Kimura, and Kodama); 2 Takuseikai Medical Corporation, Kanda Maternity Clinic, Suita, Osaka, Japan (Kawanishi); 3 Takuseikai Medical Corporation, Hanshin Birth Clinic, Amagasaki, Hyogo, Japan (Nakamura); 4 Department of Obstetrics and Gynecology, Higashiosaka City Medical Center, Higashiosaka, Osaka, Japan (Toshihiro Kimura); 5 Sakai City Medical Center, Sakai, Osaka, Japan (Tadashi Kimura)

**Keywords:** low-lying placenta, placental migration, postpartum hemorrhage, previa

## Abstract

**Background:**

Almost 10% of all pregnant women are diagnosed with placental location abnormalities (PLA), including placenta previa and low-lying placenta; however, most of PLA resolve as pregnancy process. Despite of placental migration, postpartum hemorrhage (PPH) is often experienced in those cases. The association between the timing of placental migration and the risk of PPH has remained unclear.

**Objective:**

The aim of this study is to investigate the relationship between the timing of placental migration and the PPH, and to establish a cutoff value to accurately predict the risk of PPH in cases who underwent the placental migration during course of pregnancy.

**Study Design:**

This was a retrospective cohort study using electronic medical records in Osaka University Hospital (Japan). Patients diagnosed with PLA after 22 gestational weeks using transvaginal ultrasonography were eligible for inclusion. All patients delivered at our hospital between 2009 and 2022. Focusing on the cases in which placental migration (more than 2 cm away from the internal cervical os) were observed, the association between the timing of placental migration and the PPH was investigated only in vaginal delivery using the multivariate analyses with collecting covariates that could affect the PPH. Based on the results, receiver operating characteristic curves were generated to determine the optimal cutoff for the number of weeks of placental migration to predict PPH.

**Results:**

One hundred and forty-five patients were diagnosed with PLA after 22 gestational weeks and delivered at our hospital between 2009 and 2022. Seventy-six after-placental migration cases with successful vaginal delivery were analyzed. The median gestational age of placental migration was 32 weeks, and the median amount of hemorrhage during delivery was 777 mL. PPH (500–1000 mL) occurred in 34 cases (44.7%), >1000 mL in 26 (34.2%). In the univariate analysis, a positive correlation was observed between the timing of placental migration and the amount of hemorrhage (*r*=0.365, *P*<.01). Furthermore, multivariate analysis showed that the timing of placental migration is the most strongly influential covariate (standardized partial regression coefficient: 0.31, *P*=.0036) for predicting the amount of hemorrhage. In addition, the timing of placental migration was a significant risk for PPH (more than 500 mL) (adjusted unit OR 1.26 [95% CI: 1.07–1.49]). The receiver operating characteristic curve indicated that the moderate cutoff of the timing of placental migration to predict the risk of PPH (more than 500 mL) was 29 gestational weeks. In comparison between the groups in which placental migration was confirmed before 29 gestational weeks (*n*=19) and after 30 gestational weeks (*n*=57), the incidences of PPH (more than 500 mL) were significantly higher in the group in which placental migration was confirmed after 30 gestational weeks (52.6% vs 87.7%, *P*=.0012).

**Conclusion:**

The timing of placental migration is positively correlated with the occurrence of PPH. In particular, cases in which placental migration is confirmed after 30 gestational weeks should be monitored carefully as high risk for PPH, as the risk of PPH is not eliminated.

## Highlights

• The timing of placental migration is associated with the risk of PPH
• The cutoff value of placental migration to predict PPH is 30 gestational weeks.

**AJOG Global Reports at a Glance**

**Why was this study conducted?**

The aim of this study is to investigate the relationship between the gestational weeks at which placenta previa or low-lying placenta was resolved and the postpartum hemorrhage (PPH), and to establish a cutoff value to accurately predict the risk of PPH in the patients who underwent the placental migration during course of pregnancy.

**What are the key findings?**

The positive correlation between the timing of placental migration and the amount of hemorrhage was observed. The moderate cutoff of the timing of placental migration to predict the risk of PPH was 30 gestational weeks.

**What does this study add to what is already known?**

This study adds to previous reports by demonstrating that later timing of placental migration is associated with a greater amount of postpartum hemorrhage. Especially when placental migration occurs after 30 gestational weeks, increased attention should be given to the risk of PPH.

## Introduction

Placental location abnormalities (PLA) includes placenta previa which placenta covers internal OS, and low-lying placenta (LLP) which the distance from the placental edge to the internal OS is less than 2 cm.^1^ The incidence of PLA increases with the growing number of artificial reproductive technology pregnancies.^2^ PLA is a risk factor for postpartum hemorrhage (PPH)^3^; thus, the prediction of PPH and comprehensive medical care are mandatory. In the second trimester, more than 10% of all pregnant women who undergo transvaginal ultrasound are diagnosed with PLA^4^; however, in most cases, PLA resolves as pregnancy progresses.^5^ In LLP cases where the distance from the placental edge to the internal OS is less than 1 cm, the success rate of vaginal delivery is low; therefore, vaginal delivery is generally not recommended.^6^ In contrast, in LLP cases where the distance from the placental edge to the internal OS is between 1 and 2 cm, a trial of labor can be successful in approximately 69%.^6^ However, the risk of PPH remains higher. Placental migration is diagnosed when the distance from the placental edge to the internal OS has increased by more than 2 cm. However, such cases also have a higher incidence of PPH than those who were not diagnosed with PLA during pregnancy.^7^ Not only LLP but also after-placental migration (APM) is associated with a high risk of PPH; however, the association between the timing of placental migration and the risk of PPH remains unclear.

In this study, we focused on the risk of PPH in APM cases and comprehensively analyzed the cutoff value to accurately predict the risk of PPH during delivery.

## Materials and methods

### Study design and population

We enrolled 145 patients who were diagnosed with PLA after 22 gestational weeks using transvaginal ultrasonography and delivered at our hospital between 2009 and 2022. Using electronic medical records, we extracted clinical data from patients diagnosed with PLA during pregnancy and comprehensively analyzed the association between the timing of placental migration (the distance from the placental edge to the internal OS increased by more than 2 cm) and the presence or absence of obstetric complications, such as intrapartum hemorrhage. We excluded all patients who underwent elective or emergency cesarean section. The retrospective studies like this, using medical records related to perinatal care at our institution, were reviewed and approved by the institutional ethics committee. The informed consent was obtained from patients in the form of comprehensive agreement. No company or manufacturer was involved in this study.

### Procedures

In our hospital, we perform transvaginal ultrasonography in all pregnant women at 22 to 24 gestational weeks to screen for the risk of preterm birth and abnormal placentation. In cases of abnormal placentation, we reassess the placental position using transvaginal ultrasonography approximately every 2 to 4 weeks. When the distance from the placental edge to the internal OS is less than 1 cm at approximately 35 to 36 gestational weeks, unless vaginal delivery is strongly desired, we perform a cesarean section between 36 and 37 gestational weeks.

When the distance from the placental edge to the internal OS has increased by more than 1 cm by 36 gestational weeks, we offer a trial of labor. Delivery management is based on our institution’s clinical standards, which were developed based on the practice guidelines of the American College of Obstetricians and Gynecologists.^8^ A cesarean section after a trial of labor is only performed for clinical indications such as nonreassuring fetal status, prolonged labor, and failure of vacuum and is generally not performed at the patient’s request or for social reasons. After a vaginal delivery, blood loss is recorded every few minutes using pads placed under the patient’s buttocks. Our treatment for PPH due to uterine atony is as follows: when the blood loss is (1) over 500 mL, we immediately increase oxytocin infusion and add uterotonic agents such as methylergometrine or misoprostol; (2) for over 1000 mL, we often perform intrauterine balloon tamponade and prepare blood transfusion; and (3) for over 1500 to 2000 mL, we start blood transfusion and consider interventional radiology or hysterectomy.

### Outcome measures

The main outcome was the relationship between the timing of placental migration and the amount of hemorrhage at delivery in APM cases. The amount of hemorrhage included counts up to 2 hours after delivery and excluded the estimated amount of amniotic fluid recorded in the medical record. However, if the patient was still bleeding at 50 mL/h or more at 2 hours after delivery, we added the amount of hemorrhage up to the point of bleeding control. The secondary outcomes were the relationship between PPH (more than 500 mL) and the timing of placental migration, establishment of a cutoff value to accurately predict the risk of PPH during delivery in APM cases using the receiver operating characteristic (ROC) curve, correlation between the timing of placental migration and PPH (more than 1000 mL), transfusion rate, and invasive additional procedure to control bleeding after delivery.

### Statistical analysis

All continuous variables were checked by the Shapiro-Wilk test to determine whether they were normally distributed, and a normal distribution was assumed when *P* value ≥.05. The characteristics of normally distributed variables were described as means and standard deviations, while nonparametric variables were expressed as medians and interquartile ranges. Categorical variables were presented as frequencies and percentages (%). The correlation between the timing of placental migration and the amount of hemorrhage, the primary outcome, was analyzed using Spearman’s rank-sum correlation coefficient. Covariates that could affect the amount of hemorrhage were selected based on the literature^9^, ^10^, ^11^ and clinical reasons. In multivariate analysis, the number of covariates added was set based on the number of samples. Multicollinearity was not ensured among the covariates. The relationship between the amount of hemorrhage and these covariates, including the timing of placental migration, was examined using the Wilcoxon rank-sum test. Multiple regression analysis was used to study the association between each covariate, including the timing of placental migration and the amount of hemorrhage. For the secondary outcome, we set PPH more than 500 mL as the objective variable and performed multiple logistic regression analysis using the same covariates, including the timing of placental migration. Based on these results, ROC curves were generated to determine the optimal cutoff for the number of weeks of placental migration to predict PPH. Statistical significance was defined as *P*<.05. Statistical analyses were performed using JMP Pro version 17.0.0 (SAS Institute, NC).

## Results

**Figure 1:**
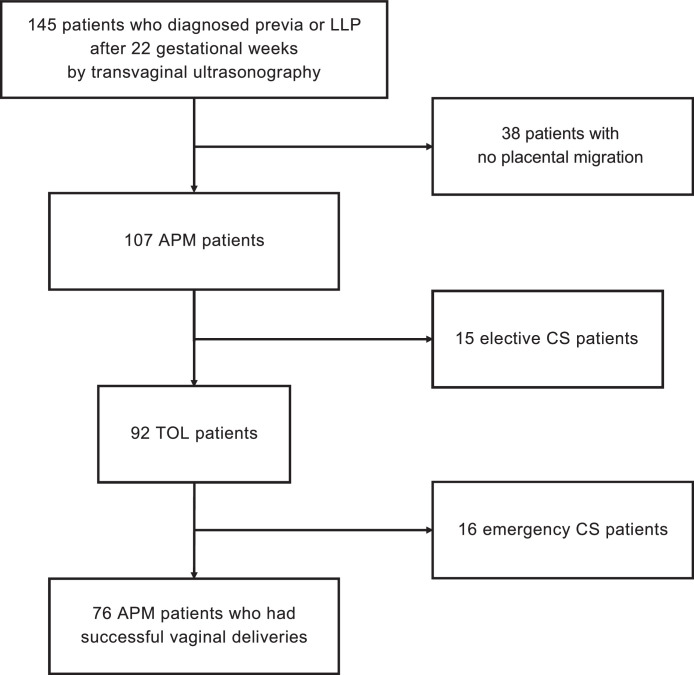
Flow chart *APM*, after-placental migration; *CS*, cesarean section; *LLP*, low-lying placenta; *TOL*, trial of labor. One hundred and forty-five patients were diagnosed previa or LLP after 22 gestational weeks and delivered in our hospital. Thirty-eight patients with no placental migration, Thirty-one patients received CS due to indications other than an abnormal placentation were excluded. Finally, Seventy-six patients with placental migration by 35 to 36 gestational had successful vaginal deliveries.

We recruited 145 patients diagnosed with PLA using transvaginal ultrasonography after 22 gestational weeks who delivered at Osaka University Hospital between 2009 and 2022. Thirty-eight patients without placental migration (placenta previa and LLP) and 31 cesarean cases (15 elective and 16 emergency) were excluded (Figure 1). Interestingly, there were no cases of emergency cesarean due to bleeding from PLA. Therefore, we analyzed 76 PAM cases with successful vaginal delivery, and the patient characteristics of those are shown in Table 1. The mean age was 35.5 years, and the median timing of delivery was 39.5 weeks. In the analysis of risk factors for PPH, in vitro fertilization (IVF) was performed in 15 cases (9.7%). Prolonged labor was observed in 16 (21.1%), vacuum extraction in 10 (13.2%), chorioamnionitis (CAM) in 2 (2.6%), heavy-for-date (HFD) in 5 (6.6%), and hypertensive disorders of pregnancy in 3 cases (3.9%). No patients with other high-risk factor of PPH, such as huge myoma, polyhydramnios, twin pregnancy, a history of severe PPH, were observed. The median gestational age at the time of placental migration was 32 weeks, and the median amount of hemorrhage during delivery was 777 mL. In PPH incidence, the blood loss was 500 to 1000 mL in 34 cases (44.7%), >1000 mL in 26 (34.2%), and transfusion in 8 (10.5%). Owing to PPH, intrauterine balloon tamponade was performed in 5 (6.6%) and hysterectomy in 1 (1.3%). In 76 APM cases, obvious neonatal distress (eg, umbilical cord artery blood pH <7.1 or Apgar score ≦6) was not observed.

Yamamoto et al. Placenta migration after 30 gestational weeks is a risk for postpartum hemorrhage. AJOG Glob Rep 2025.

**Table 1:** Patient characteristics

Characteristic	APM patients with successful vaginal deliveries (*n*=76)
Age (y)	35.5±5.0^a^
Gravidity/parity	2 (1–3)/0 (0–2)
BMI (kg/m^2^)	21.3 (19.7–23.3)
IVF	15 (19.7)
Anterior placenta	14 (18.7)
Timing of delivery (wk)	39.5 (39–40)
Prolonged second stage of labor^b^	16 (21.1)
Vacuum extraction	10 (13.2)
Chorioamnionitis^c^	2 (2.6)
HFD^d^	5 (6.6)
Epidural labor	19 (25.0)
HDP	3 (3.9)
Timing of placental migration (wk)^e^	32 (29–35)
Amount of bleeding in delivery (mL)	777 (523–1229)
PPH (500–1000 mL)	34 (44.7)
PPH (>1000 mL)	26 (34.2)
Blood transfusion	8 (10.5)
IUBT	5 (6.6)
Hysterectomy	1 (1.3)
Birth weight (g)	3089±344^a^
Umbilical cord artery blood pH	7.30 (7.26–7.33)

Data are presented as numbers (percentages) or medians (interquartile ranges), unless otherwise indicated.

*APM*, after-placental migration; *BMI*, body mass index; *CS*, cesarean section; *HDP*, hypertensive disorders of pregnancy; *HFD*, heavy-for-date; *IUBT*, intrauterine balloon tamponade; *IVF*, in vitro fertilization; *PPH*, postpartum hemorrhage.

a Data are presented as mean (standard deviation).

b Defined as the second stage of labor being 3 hours or longer for primipara (4 hours or longer for epidural labor) and 2 hours or longer for multipara (3 hours or longer for epidural labor).

c Use diagnostic criteria for clinical chorioamnionitis according to Lencki et al.

d Birth weight in gestational weeks at or above the 90th percentile.

e Gestational weeks in which migration was confirmed by transvaginal ultrasonography.

**Figure 2:**
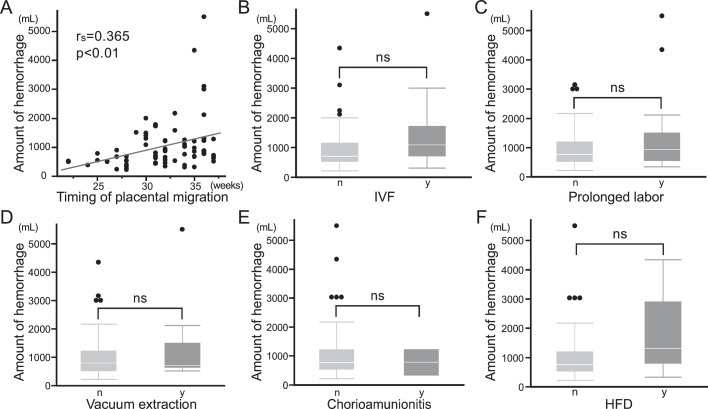
Relationship between the amount of hemorrhage and each variable (univariate analysis) *IVF*, in vitro fertilization; *HFD*, heavy-for-date.(A) Correlation between the timing of placental migration (gestational weeks) and the amount of hemorrhage. Each dot represents an individual case. rs indicates Spearman's rank correlation coefficient. (B-F) Comparison of the amount of hemorrhage according to the presence or absence of (B) in vitro fertilization (IVF), (C) prolonged labor, (D) vacuum extraction, (E) chorioamnionitis, and (F) HFD. Box plots represent the median (center line), interquartile range (box), and range (whiskers) excluding outliers (dot). Statistical significance was evaluated using univariate analysis; p values are shown in the figure. ns indicates not significant.

In the 76 APM cases, a positive correlation was observed between the timing of placental migration and the amount of hemorrhage (*r*=0.365, *P*<.01) (Figure 2, A). However, other risk factors for PPH, including IVF, prolonged delivery, vacuum extraction, CAM, and HFD, were not correlated with the amount of hemorrhage in the univariate analysis (Figure 2, B–F). Of 76 APM cases in this study, 41 had no risk factors for PPH. Moreover, the analysis that focused on these 41 APM cases showed a positive correlation between the timing of placental migration and the amount of hemorrhage (data not shown).

Yamamoto et al. Placenta migration after 30 gestational weeks is a risk for postpartum hemorrhage. AJOG Glob Rep 2025.

Next, using multivariate analysis, we investigated whether these covariates, including the timing of placental migration, IVF, prolonged delivery, vacuum extraction, CAM, and HFD, affected the amount of hemorrhage (Table 2). The analysis of the variance model showed a significant result (*P*=.0010), indicating that this model was useful as a predictive formula for PPH. The partial regression coefficients for each variable showed that the timing of placental migration, IVF, and HFD were risk factors for hemorrhage. As the timing of placental migration was delayed by 1 week, the amount of hemorrhage increased by 72.8 mL. Moreover, in IVF cases, the amount of hemorrhage increased by 282.5 mL; in HFD cases, it increased by 421 mL (Table 2). The standard partial regression coefficients for the timing of placental migration, IVF, and HFD were 0.31, –0.25, and –0.24, respectively. Multicollinearity was checked to confirm the absence of strong correlations among the covariates. These findings indicate that the timing of placental migration is the most strongly influential covariate for predicting the amount of hemorrhage. In addition, the timing of placental migration was a significant risk factor for PPH (more than 500 mL) (odds ratio: 1.26 [95% confidence interval: 1.08–1.48], and the adjusted unit odds ratio with multivariate analysis was 1.26 [95% confidence interval: 1.07–1.49]) (Table 3).

**Table 2:** Relationship between the amount of hemorrhage and each variable (multivariate analysis)

Variable	*B*	SE	β	*P* value
Timing of placental migration	72.8	24.1	0.31	.0036
IVF	–282.5	116.4	–0.25	.0018
Prolonged second stage of labor	–185.3	118.9	–0.17	.12
Vacuum extraction	–67.8	144.0	–0.05	.64
Chorioamnionitis	287.4	303.6	0.10	.35
HFD	–421.0	196.0	–0.24	.036

Analysis of the variance model, *P*=.0010, adjusted *R*^2^=0.27.

β, standardized partial regression coefficient; *B*, partial regression coefficient; *HFD*, heavy-for-date; *IVF*, in vitro fertilization; *SE*, standard error.

**Table 3:** Relationship between PPH (more than 500 mL) incidence and variables (multivariate analysis)

Variable	PPH+ (*n*=60)	PPH– (*n*=16)	OR (95% CI)	*P* value^a^	Adjusted OR (95% CI)	*P* value^a^
Timing of placental migration	-	-	1.26^b^ (1.08–1.48)	.0036	1.26^b^ (1.07–1.49)	.0062
IVF	13 (21.7)	2 (12.5)	1.94 (0.39–9.63)	.42	1.69 (0.28–10.1)	.56
Prolonged second stage of labor	14 (23.3)	2 (12.5)	2.13 (0.43–10.5)	.35	1.80 (0.28–11.4)	.53
Vacuum extraction	10 (16.7)	0 (0)	-	-	-	-
Chorioamnionitis	1 (1.7)	1 (6.3)	0.25 (0.015–4.30)	.34	0.27 (0.0098–7.46)	.44
HFD	4 (6.7)	1 (6.3)	1.07 (0.11–10.3)	.95	1.83 (0.097–34.3)	.69

Model chi-square test *P*=.016; Hosmer–Lemeshow test *P*=.48.

*CI*, confidence interval; *HFD*, heavy-for-date; *IVF*, in vitro fertilization; *OR*, odds ratio.

a Wald test.

b Unit odds ratio.

**Figure 3:**
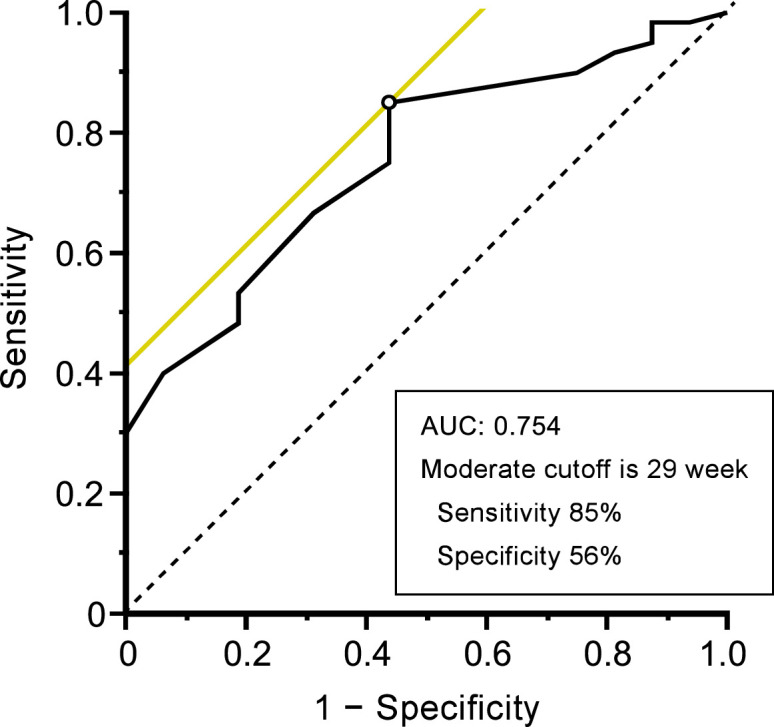
ROC analysis to examine the moderate timing of placental migration for predicting PPH (more than 500 mL) incidence

Moreover, ROC curves were generated based on the results of the univariate analysis. The ROC curve indicated that the moderate cutoff of the timing of placental migration to precisely predict the risk of PPH (more than 500 mL) was 29 gestational weeks (sensitivity, 85%; specificity, 56%; AUC, 0.754) (Figure 3).

Yamamoto et al. Placenta migration after 30 gestational weeks is a risk for postpartum hemorrhage. AJOG Glob Rep 2025.

In addition, we compared patient characteristics between the groups in which placental migration was confirmed before 29 gestational weeks (*n*=19) and after 30 gestational weeks (*n*=57). The amounts of bleeding and the incidence of PPH were significantly higher in the group in which placental migration was confirmed after 30 gestational weeks (Table 4). Notably, most patients who have PPH (more than 1000 mL) were in the group with the placental migration after 30 gestational weeks (Table 4).

**Table 4:** Patient characteristics (migration before 29 weeks vs migration after 30 weeks)

Characteristic	APM patients with placental migration before 29 gestational weeks (*n*=19)	APM patients with placental migration after 30 gestational weeks (*n*=57)	*P* value
Age (y)	35.4±4.9^a^	35.5±5.1^a^	.93
Gravidity/parity	2 (1–3)/1 (0–1)	2 (1–3)/0 (0–1)	.60/.57
BMI (kg/m^2^)	22.6 (20.3–25.1)	20.8 (19.3–23.1)	.06
IVF	2 (10.5)	13 (22.8)	.24
Anterior placenta	4 (21.1)	10 (17.9)	.76
Timing of delivery (wk)	39 (38–40)	40 (39–40)	.31
Prolonged second stage of labor^b^	3 (15.8)	13 (22.8)	.52
Vacuum extraction	2 (10.5)	8 (14.0)	.70
Chorioamnionitis^c^	1 (5.2)	1 (1.8)	.41
HFD^d^	1 (5.3)	4 (7.0)	.79
Epidural labor	4 (21.1)	15 (26.3)	.65
HDP	0 (0.0)	3 (5.3)	.31
Timing of placental migration (wk)^e^	27 (25–28)	34 (32–36)	<.0001
Amount of bleeding in delivery (mL)	512 (346–670)	881 (661–1300)	<.001
PPH (>500 mL)	10 (52.6)	50 (87.7)	.0012
PPH (>1000 mL)	1 (5.3)	25 (43.9)	.0021
Blood transfusion	0 (0.0)	8 (14.0)	.08
IUBT	0 (0.0)	5 (8.8)	.18
Hysterectomy	1 (5.3)	0 (0.0)	.08
Birth weight (g)	3074±367^a^	3094±339^a^	.83
Umbilical cord artery blood pH	7.31 (7.27–7.33)	7.29 (7.26–7.34)	.47

Data are presented as numbers (percentages) or medians (interquartile ranges), unless otherwise indicated.

*APM*, after-placental migration; *BMI*, body mass index; *CS*, cesarean section; *HDP*, hypertensive disorders of pregnancy; *HFD*, heavy-for-date; *IUBT*, intrauterine balloon tamponade; *IVF*, in vitro fertilization; *PPH*, postpartum hemorrhage.

a Data are presented as mean (standard deviation).

b Defined as the second stage of labor being 3 hours or longer for primipara (4 hours or longer for epidural labor) and 2 hours or longer for multipara (3 hours or longer for epidural labor).

c Use diagnostic criteria for clinical chorioamnionitis according to Lencki et al.

d Birth weight in gestational weeks at or above the 90th percentile.

e Gestational weeks in which migration was confirmed by transvaginal ultrasonography.

Finally, we performed multivariate analysis to analyze the relationship between the timing of placental migration and other outcomes (Table 5). The timing of placental migration was a significant factor in PPH (more than 1000 mL), and the adjusted odds ratio was 1.21 (95% confidence interval: 1.04–1.46). However, no clear association was observed between the timing of placental migration and other outcomes, including blood transfusion rate and invasive procedures to control PPH.

**Table 5:** Relationship between timing of placental migration and other outcomes (multivariate analysis)

Outcome	OR (95% CI)	*P* value^a^	Adjusted OR (95% CI)	*P* value^a^	Model chi-square test *P* value
PPH (≧1000 mL)	1.17 (1.02–1.37)	.03	1.21 (1.04–1.46)	.025	.016
Transfusion rate	1.26 (0.97–1.65)	.09	1.23 (0.94–1.61)	.12	.34
Invasive additional procedures^b^	1.15 (0.95–1.42)	.15	1.14 (0.93–1.40)	.22	.25
Drainage	1.29 (0.88–1.91)	.13	1.30 (0.87–1.92)	.20	.45
IUBT	1.24 (0.90–1.7)	.14	1.22 (0.88–1.69)	.24	.67
Hysterectomy	0.69 (0.39–1.20)	.14	0.03 (not estimable^c^)	.99	.10
Bilateral compression	1.10 (0.95–1.26)	.47	1.07 (0.93–1.22)	.35	.50
Misoprostol (anal insertion)	1.09 (0.95–1.26)	.20	1.12 (0.97–1.30)	.12	.42
Methylergometrine (intramuscular)	1.13 (0.97–1.32)	.10	1.17 (0.99–1.40)	.047	.18

*CI*, confidence interval; *HFD*, heavy-for-date; *IVF*, in vitro fertilization; *OR*, odds ratio.

a Wald test.

b Include drainage of intrauterine coagulation, IUBT, and hysterectomy.

c CI not estimable due to sparse event counts and quasi-complete separation.

## Discussion

### Principal findings

In our study, we revealed that the timing of placental migration is significantly associated with the amount of hemorrhage and the occurrence of PPH. Moreover, we proposed 30 gestational weeks as the new standard for risk assessment of PPH in APM cases.

### Results in the context of what is known

A previous report has shown that APM is associated with a higher risk of PPH than normal placentation throughout pregnancy.^7^ Therefore, the Japan Society of Obstetrics and Gynecology guidelines recommend a diagnosis of abnormal placentation after 20 gestational weeks and reassessment at 32 gestational weeks. The Royal College of Obstetricians and Gynecologists also recommends reassessing placental sites at 32 gestational weeks for referral to tertiary care facilities.^12^ However, statistical data exist regarding the timing of migration and the risk of PPH, and each guideline only has recommendations regarding the timing of referring patients to tertiary care facilities. Our study showed that in APM cases, the incidence of PPH (more than 500 mL) increased with the timing of placental migration. Moreover, we demonstrated statistical evidence of the relationship between the timing of placental migration and the risk of PPH using univariate and multivariate analyses. We selected the covariates that affect the outcome of PPH appropriately in consideration of the sample size.^9^, ^10^, ^11^ Our study provides crucial data that can influence guidelines.

### Clinical implications

Previous reports have shown that PLA, including previa and LLP, is identified in 10.6% of women undergoing screening for placental position at 22 to 24 gestational week.^4^ Although 90% of cases of abnormal placentation diagnosed before 20 gestational weeks are resolved by delivery,^5^^,^^13^ APM may account for 10% of all pregnancies. APM is rarely reported and is not fully recognized as a risk factor for PPH^3^; thus, APM should be recognized as a hidden risk factor for PPH.

### Research implications

According to one theory, the placenta migrates because of the elongation of the lower uterine segment.^14^ Another theory, the trophotropism theory, suggests that the placenta grows preferentially toward the fundus in search of a richer blood supply than the lower part of the uterus, which has a poor vascular structure. This theory has been proposed as the mechanism of placental migration.^15^^,^^16^ Based on these findings, early placental formation in the lower segment of the uterus may result in increased vascularity in the lower uterine segment, which should have fewer vascular structures. In addition, the placenta remains in the lower part of the uterus until later gestational weeks, forming a more developed vascular structure. Owing to the inadequate contraction of these vascular beds, PPH occurs more frequently in APM cases. Furthermore, an abnormal placental position is difficult to resolve if the rate of placental migration is not smooth after the third trimester,^17^ and the lower the placental position at delivery, the greater the amount of hemorrhage after delivery.^6^^,^^18^ Therefore, it was hypothesized that a prolonged abnormal placental position indicates that the placenta forms in the lower position of the uterus and may increase the amount of hemorrhage after delivery. The results of this study are consistent with this hypothesis.

### Strengths and limitations

This is a first study to showed the association between the timing of placental migration and the risk of PPH, and also showed that the cutoff value of placental migration to predict PPH is 30 gestational weeks. The relevant covariates that may influence the outcome of PPH were appropriately collected, and the statistical analyses were conducted accordingly.

This study has some limitations. First, this was a retrospective study conducted in a single institution, and we did not follow up on all APM cases diagnosed in our hospital. Some APM patients returned to the primary clinic or hospital, and collecting clinical information after delivery was challenging. A larger study on patients who returned to the primary facility should be conducted to confirm our findings.

Second, we did not compare APM with normal cases. APM cases should be compared with normal cases during the same observation period to evaluate the risk of PPH in APM cases. However, the definition of PPH has been established as a cutoff of 500 mL; thus, this may not be strictly necessary.

## Conclusions

In conclusion, our study revealed that the timing of placental migration is positively correlated with the occurrence of PPH. In particular, cases in which placental migration is confirmed after 30 gestational weeks should be monitored carefully after delivery as high risk for PPH, as the risk of PPH is not eliminated.

## CRediT authorship contribution statement

**Gaku Yamamoto:** Writing – review & editing, Writing – original draft, Resources, Methodology, Investigation, Formal analysis, Data curation. **Kosuke Hiramatsu:** Writing – review & editing, Writing – original draft, Validation, Project administration, Methodology, Investigation, Data curation, Conceptualization. **Yoko Kawanishi:** Investigation. **Mamoru Kakuda:** Investigation. **Koji Nakamura:** Investigation. **Tatsuya Miyake:** Investigation. **Kazuya Mimura:** Writing – review & editing, Supervision, Investigation. **Toshihiro Kimura:** Investigation. **Masayuki Endo:** Supervision, Investigation. **Tadashi Kimura:** Supervision, Investigation. **Michiko Kodama:** Supervision, Investigation.
